# Vogt–Koyanagi–Harada disease after SARS‐CoV‐2 infection: Case report and literature review

**DOI:** 10.1002/iid3.1250

**Published:** 2024-04-25

**Authors:** Hui Zou, Ke Zhang, Xuan Chen, Sha Sha

**Affiliations:** ^1^ Department of Ophthalmology Hunan Provincial People's Hospital (The First Hospital of Hunan Normal University) Changsha Hunan China

**Keywords:** case report, COVID‐19, SARS‐CoV‐2, Vogt–Koyanagi–Harada disease

## Abstract

**Background:**

Severe acute respiratory syndrome coronavirus 2 (SARS‐CoV‐2) is responsible for coronavirus disease 2019 (COVID‐19), a complex and multifaceted illness. COVID‐19 is associated with various ocular manifestations including conjunctivitis, retinal vein occlusion and optic neuritis. However, the case of Vogt‐Koyanagi‐Harada (VKH) disease associated with SARS‐CoV‐2 is infrequent, and the specific association is still unclear.

**Case Presentation:**

In the present study, a 35‐year‐old female patient without any significant medical history presented with 1 week of bilateral blurred vision, occurring 2 weeks after a clinical course of COVID‐19. Upon examination, both eyes exhibited bullous serous retinal detachments. She was diagnosed with incomplete VKH disease. Early diagnosis and treatment of VKH disease are essential for the visual prognosis of this aggressive disease. In this particular patient, ocular inflammatory signs and visual acuity improved via corticosteroid therapy. It is worth noting that the occurrence of VKH disease associated with SARS‐CoV‐2 is uncommon, and the specific connection between the two remains unknown. We review and summarize the clinical characteristics of VKH disease following SARS‐CoV‐2 infection, and discuss the potential mechanisms that may explain this phenomenon, based on similar studies previously reported.

**Conclusion:**

Despite the unclear causality, it is important for ophthalmologists and physicians to be recognizant of the possible association between VKH disease and COVID‐19. SARS‐CoV‐2 may play a potential immunological triggering role in VKH disease. However, further in‐depth research is necessary to investigate the clinical and epidemiological features, as well as the underlying mechanisms of this association.

## Graphical abstract

we report a 35‐year‐old female patient developed incomplete Vogt–Koyanagi–Harada (VKH) disease 2 weeks after a clinical course of COVID‐19. Severe acute respiratory syndrome coronavirus 2 may play a potential immunological triggering role in VKH disease.

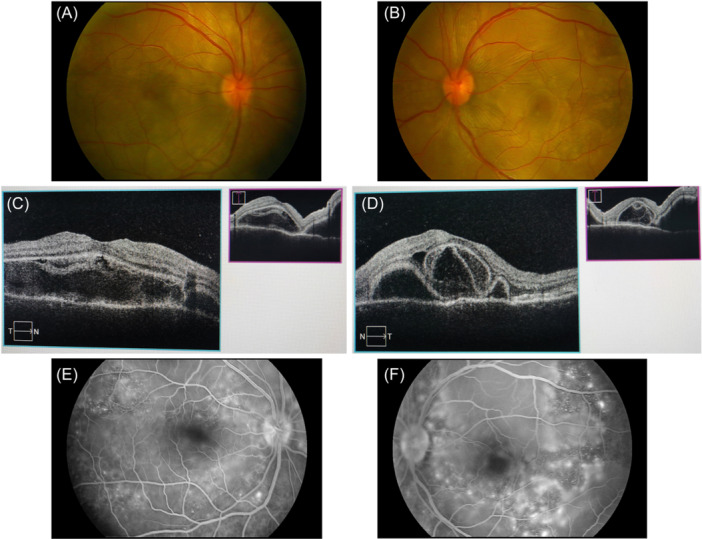

## 1 INTRODUCTION

Vogt‐Koyanagi‐Harada disease (VKH) is not only an autoimmune vision‐threatening disease that is frequently characterized by bilateral granulomatous panuveitis, but also a multisystemic inflammatory disorder often accompanied by neurological, auditory, and integumentary symptoms, such as headache, tinnitus, deafness, alopecia, and so on. Although the etiology and pathogenesis of VKH are unknown, considerable progress has been made on the subject in recent decades. Numerous hypotheses have been proposed, primarily focusing on genetic predisposition and viral infections. It is now widely accepted that viruses may trigger an autoimmune response to melanin through the mechanism of molecular mimicry in VKH disease. Upon reviewing the available studies, it has been observed that various infectious triggers, such as Epstein‐Barr virus (EBV) and cytomegalovirus (CMV), have been implicated in the onset of VKH disease.^1^

The emergence of severe acute respiratory syndrome coronavirus‐2 (SARS‐CoV‐2), the causative pathogen of the coronavirus disease 2019 (COVID‐19) outbreak, has led to reports of ocular manifestations associated with COVID‐19, most of which are conjunctivitis, retinal vein occlusion and optic neuritis.^2^, ^3^ However, it is worth noting that the case of VKH disease associated with SARS‐CoV‐2 is rare, and the specific association is still unclear. Thus, this study presents a case of bilateral panuveitis resembling VKH disease following SARS‐CoV‐2 infection and conducts a comprehensive review of current pertinent literature to analyze some potential interactions between SARS‐CoV‐2 and VKH disease. This patient provided her written informed consent to participate in this study.

## 2 CASE REPORT

A 35‐year‐old female patient with no significant medical history presented to our ophthalmic outpatient department with 1 week of bilateral blurred vision. She reported experiencing fever, headache, and cough 2 weeks before her visit, during which she tested positive for SARS‐CoV‐2 by polymerase chain reaction (PCR). She had no history of ocular trauma or surgery before the occurrence of uveitis.

**Figure 1:**
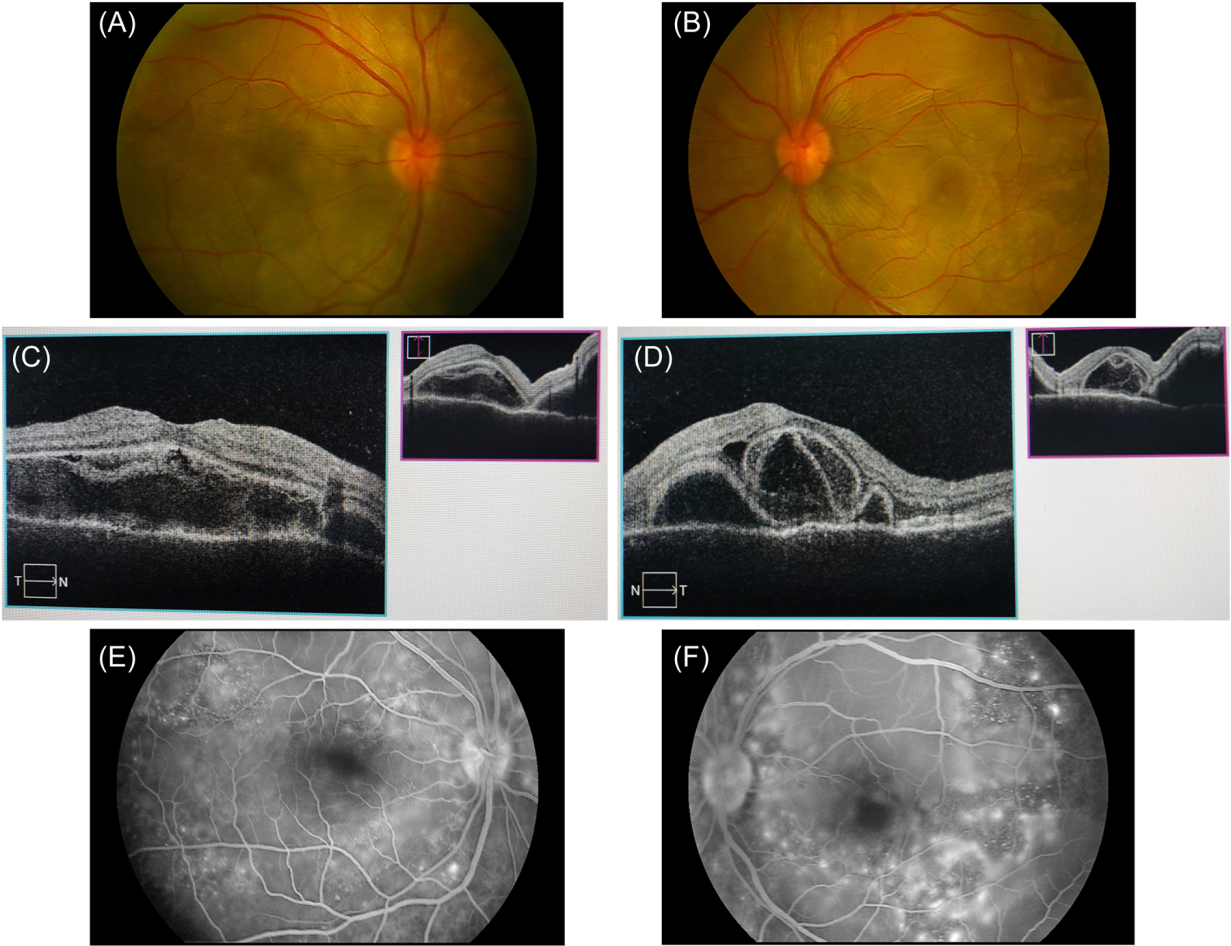
Fundus findings of panuveitis at initial presentation. Color fundus photographs showing bullous serous retinal detachment (SRD) in the posterior retina and optic disc hyperemia in the right eye (A) and the left eye (B). Optical coherence tomography (OCT) images revealing SRDs, cystoid spaces in the neurosensory layer of the retina and bacillary layer detachment in the right eye (C) and the left eye (D). Fluorescein angiography images indicating multiple punctate fluorescein leaks and late pooling of the dye consistent with the SRD locations, and optic disc staining in the right eye (E) and the left eye (F).

**Figure 2:**
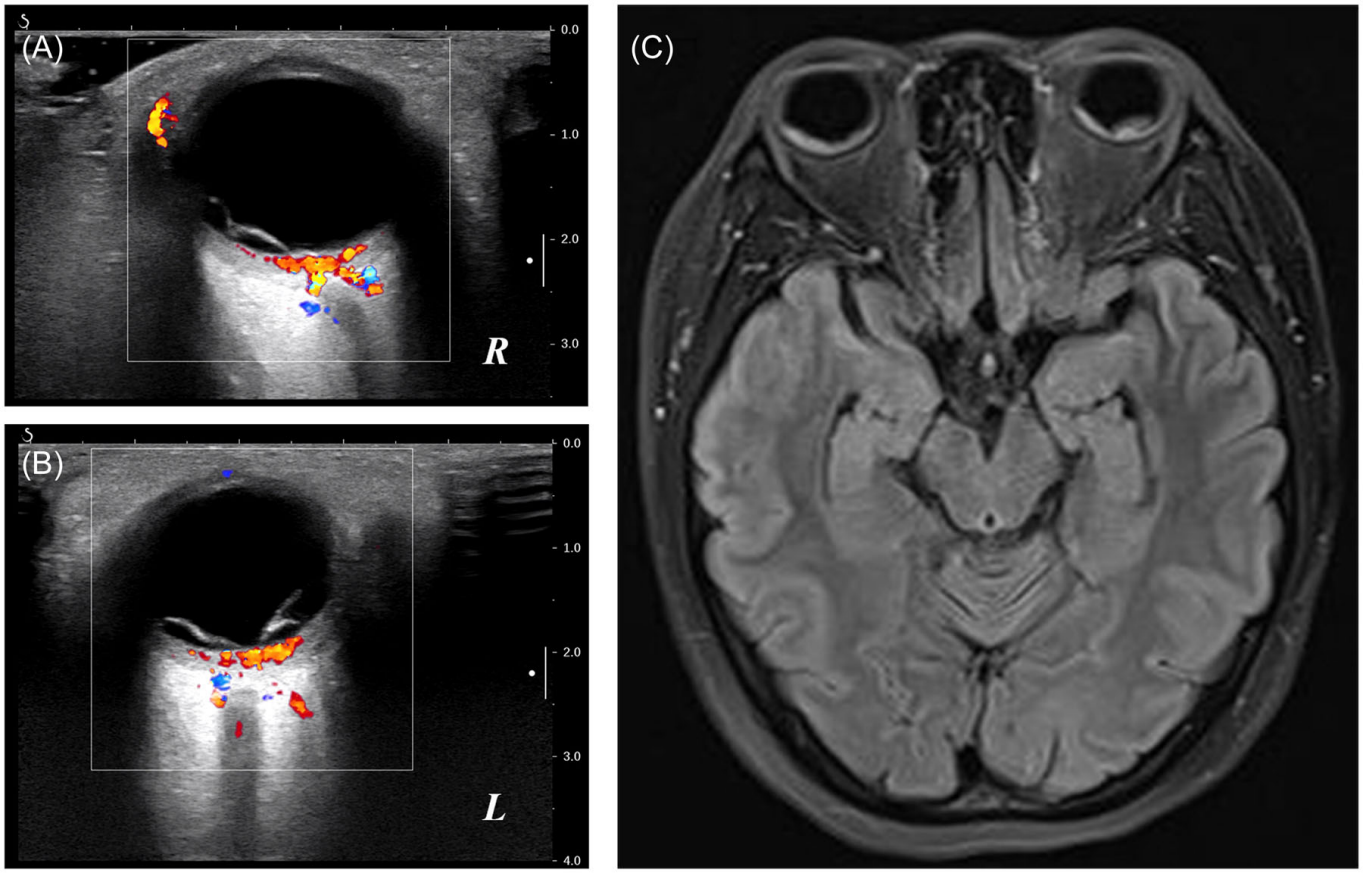
B‐scan ultrasonography findings highlighting serous retinal detachment and thickened choroid in the right eye (A) and the left eye (B). Brain MRI images (C) demonstrating bilateral retinal detachment. MRI, magnetic resonance imaging.

Ophthalmological examinations showed that her best corrected visual acuity was 20/125 in the right eye and 20/100 in the left eye. Intraocular pressure was 21 mmHg in the right eye and 22 mmHg in the left eye. Upon examination under a slit lamp, no keratic precipitates or flares were detected in the anterior segment, while vitreous opacities were grade 1+ inflammatory cells in both eyes. Funduscopic examination showed bullous serous retinal detachments (SRD) with subretinal fluid in the posterior retina of both eyes (Figure 1A,B). Furthermore, optical coherence tomography (OCT), B‐scan ultrasonography and fundus fluorescein angiography (FFA) were also performed. OCT provided further insights into the SRDs, revealing the presence of cystoid spaces in the neurosensory layer of the retina, which was divided into several compartments by subretinal septa. OCT also demonstrated the presence of retinal pigment epithelium folds and bacillary layer detachment (Figure 1C,D). FFA indicated multiple punctate fluorescein leakages and pooling of the dye in areas of SRDs, and optic disc hyperfluorescence (Figure 1E,F). B‐scan ultrasonography confirmed the presence of SRD and thickening of the posterior choroid in both eyes, while ruling out any evidence of posterior scleritis (Figure 2A,B).

On admission, her body temperature was 36.6 degrees Celsius (°C), and her chest X‐ray image showed no abnormal shadow. Furthermore, she was tested by PCR and was negative for SARS‐CoV‐2. She did not notice tinnitus or hair loss. A complete systemic workup was conducted. Brain magnetic resonance imaging demonstrated bilateral retinal detachment, while there was no evidence of optic nerve thickening or any intracranial or orbital space‐occupying lesion (Figure 2C). Complete blood tests were performed, reporting unremarkable outcomes. The patient's erythrocyte sedimentation rate was 20 mm/h, and the C‐reactive protein level was 0.73 mg/L. Serological tests for toxoplasmosis, EBV, CMV, Rubella virus, Herpes simplex virus 1, and HIV were negative. In addition, extensive blood tests for underlying autoimmune etiologies were carried out, all within normal limits.

**Figure 3:**
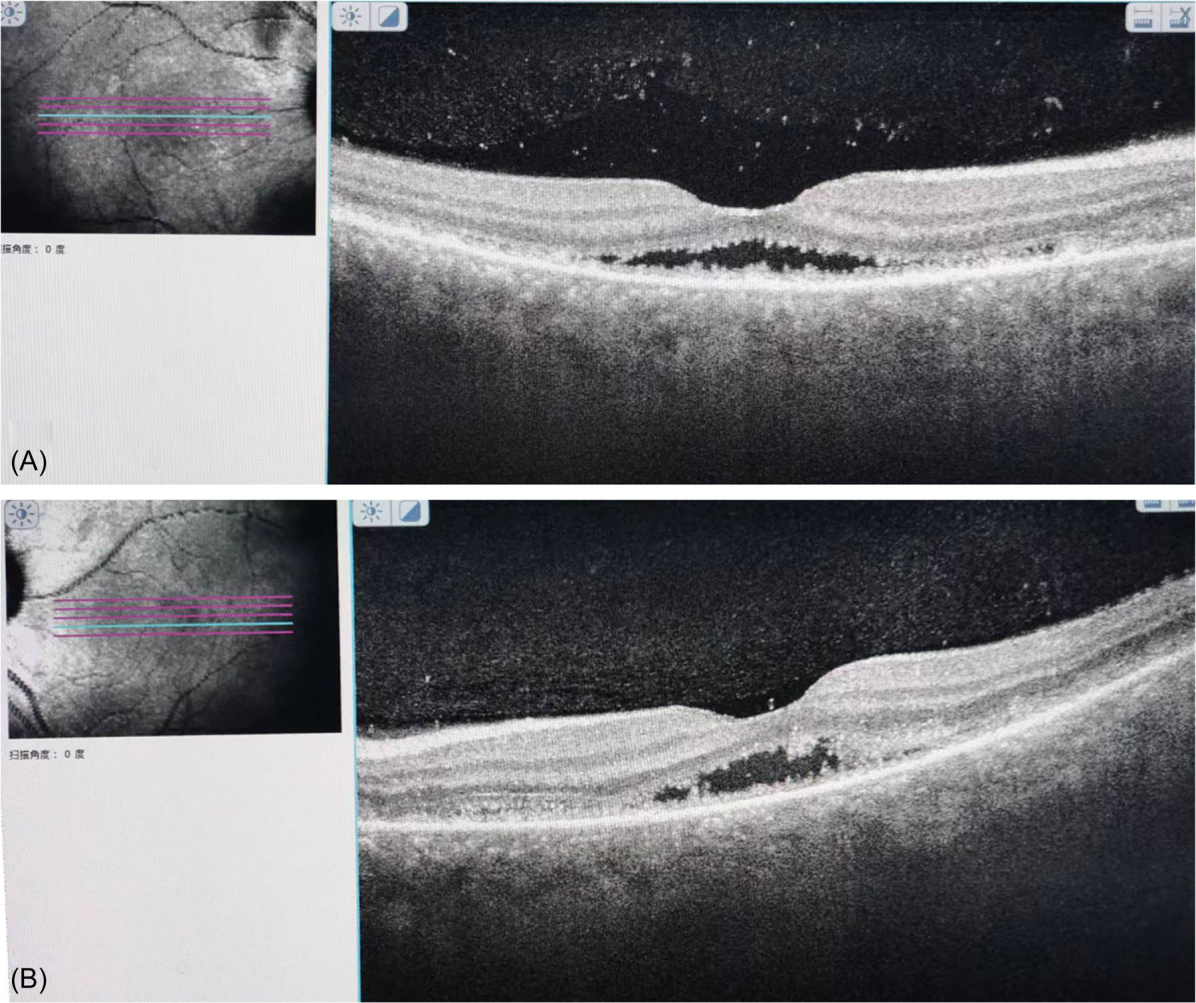
OCT of the right (A) and left (B) eye showing improvement of the serous retinal detachment and bacillary layer detachment.

According to the clinical findings and laboratory data, she was diagnosed with incomplete VKH disease due to the presence of ocular signs and neurological findings.^4^ Early and aggressive systemic glucocorticosteroid therapy remains the primary treatment according to the standard treatment for VKH disease.^5^ The patient was treated with pulse intravenous methylprednisolone therapy (1000 mg/day) followed by a subsequent high‐dose oral corticosteroid regimen upon confirmation of the VKH disease diagnosis. In the clinical course of treatment, we paid attention to the deterioration of visual acuity and SRD, and tapered the dose of prednisolone slowly. Two weeks after the first evaluation, the patient presented with an improvement in her visual acuity, measuring 20/60 in the right eye and 20/80 in the left eye. The control OCT findings revealed a significant resolution of subretinal fluid in both eyes (Figure 3).

## 3 DISCUSSION

VKH disease is a severe bilateral granulomatous panuveitis that is frequently associated with a series of systemic and ocular manifestations. Clinically, it manifests in four stages: prodromal stage, acute stage, convalescent stage, and chronic recurrent stage. The prodromal phase is characterized by flu‐like symptoms, such as fever, headache, nausea, tinnitus, and periorbital pain. In this patient, extraocular manifestations, including fever and headache, had resolved by the time of examination. Following the prodromal phase, bilateral posterior uveitis occurs, characterized by multiple exudative retinal detachments, optic disc swelling and choroidal thickening. Our patient presented with typical signs, in which inflammation was limited to the posterior segment of the eye. Based on Revised Diagnostic Criteria for VKH disease,^4^ our patient was diagnosed with incomplete VKH disease.

Studies have indicated that the clinical manifestations of VKH disease are caused by an inflammatory autoimmune response, facilitated by CD4^+^ cytotoxic T‐lymphocytes that target melanocyte‐related antigens within affected organs, such as the eye, inner ear, meninges, and skin.^1^, ^6^ VKH‐derived lymphocytes recognize synthetic peptides derived from tyrosinase, an enzyme uniquely expressed by melanocytes and involved in melanin synthesis, thereby prompting activation and initiation of the immune response in VKH disease. Furthermore, it is widely accepted that genetic factors and exogenous or environmental triggers may exert significant influence in the initial stage of VKH disease. Although the definitive mechanisms involved need further detailed research, the widely accepted hypothesis suggests that viruses may play a triggering role in the development of this disease. The presence of meningeal manifestations such as fever, headache, and the detection of EBV DNA in the cerebrospinal fluid^7^ or vitreous^8^ from patients with VKH disease, provided supporting evidence for the triggering role of viral infection in the pathogenesis. However, conflicting findings from other studies and the ubiquity of EBV in humans cast doubt on the exclusive role of this virus in the disease. According to evidence accumulated during past decades with the rapid advancement in several domains of basic science, a mechanism of molecular mimicry was proposed. Exogenous antigens encoded by viruses may resemble proteins from pigmented cells, thereby eliciting an immune response upon recognition by specific HLA‐class II molecules. Sugita et al. found similarity between a CMV‐associated peptide and tyrosinase peptide and suggested that some T cells from patients with VKH disease responded strongly to both peptides.^9^, ^10^ Moreover, microbial immune products can be recognized by Toll‐like receptors (TLRs), consequently triggering innate immune responses. Notably, the increased expression of TLR3 and TLR4 in macrophages from active VKH patients, in comparison to control subjects, provides compelling evidence for the involvement of TLRs in the pathogenesis of VKH disease.^11^

According to early studies, *Mycoplasma pneumoniae* ^12^ and influenza A virus^13^ have been reported to be associated with the development of VKH disease. In our report, this patient presented with VKH symptoms 2 weeks after SARS‐CoV‐2 infection. Thus, we hypothesized that SARS CoV‐2 may play a triggering role in initiating VKH disease.

COVID‐19 typically manifests as an acute respiratory disease characterized by inflammatory and vascular complications caused by SARS‐CoV‐2. However, SARS‐CoV‐2 is known to cause various clinical symptoms in multiple organ systems, including the respiratory system, neurological system, cardiovascular system, gastrointestinal tract, immune system, and eye.^14^ Most patients initially present with fever, headache, cough, sore throat, and fatigue. In more severe cases, COVID‐19 can progress to acute respiratory distress syndrome, cytokine storm and multiorgan failure.^15^ Our patient, confirmed to have SARS‐CoV‐2 infection by PCR, presented with fever, cough, and headache, and she presented with VKH symptoms 2 weeks after COVID‐19 infection onset.

A growing number of studies have indicated that ocular manifestations can either preexist or occur as a result of SARS‐CoV‐2 infection.^16^, ^17^ Aggarwal reported that ocular manifestations were observed in 11.64% of COVID‐19‐infected patients in a meta‐analysis.^18^ Conjunctivitis appears to be the most common ocular pathology, but there is also evidence linking COVID‐19 to retinal vascular occlusions, optic neuritis and uveitis.^2^, ^16^, ^19^ To the best of our knowledge, there have been some cases of VKH disease after COVID‐19 vaccination reported.^20^, ^21^, ^22^ However, the number of reported cases associating VKH disease with SARS‐CoV‐2 is limited to only five.^23^, ^24^, ^25^, ^26^, ^27^, ^28^ For the first time, Santamaria et al. described the possible associations between SARS‐CoV‐2 infection and VKH disease.^24^ Eatz and Charles reported a case of VKH disease 2 weeks after COVID‐19 infection onset and suggested that SARS‐CoV‐2 may be an immunological trigger of VKH if the onset of COVID‐19 infection preceded the onset of VKH symptoms by 2 weeks or occurred during the prodromal VKH phase.^23^

Table 1 presents the clinical characteristics of our case in comparison to previously reported cases of VKH disease in temporal association with COVID‐19 infection. Among the 5 cases, there was 1 male and 4 females, with ages ranging from 23 to 37 years. The onset of VKH symptoms following SARS CoV‐2 infection ranged from 2 to 3 weeks. Choroiditis severity and progression influenced vision acuity (VA), which ranged from light perception to 20/30. Oral steroids were administered as the fundamental treatment in all cases. Despite not recovering back to normal, visual acuity improved well in four patients with prompt treatment. While VA in one eye of a 32‑year‑old female did not show improvement. Our patient was also a young woman, and involvement was bilateral. The onset of symptoms occurred 2 weeks after COVID infection, and the patient received intravenous steroids as part of the initial pulse therapy, resulting in improved vision due to a favorable response to systemic corticosteroid treatment.

**Table 1:** Clinical characteristics of our case in comparison with the other previously reported patients with Vogt–Koyanagi–Harada (VKH) disease following COVID‐19.^23^, ^24^, ^25^, ^26^, ^27^

Study	Age/Sex	Vision acuity	Interval	Ocular signs	Systemic findings	Treatment	Recovery
Current case	35/F	OD: 20/125 OS: 20/100	2 weeks	OU: vitritis, optic disk hyperemia, serous RD, BLD	No	Methylprednisolone pulses (3 days), oral prednisolone	OD: 20/60 OS: 20/80
Eatz et al.^23^	27/M	OU: light perception	2 weeks	OU: vitritis, serous RD	Tinnitus	Oral prednisone and methotrexate	OD: 20/60 OS: 20/80
Santamaria et al.^24^	32/F	OU: hand motion	2 weeks	OU: anterior uveitis, vitritis, edematous and hyperemic papilla, serous RD	Headache, nausea, vomiting and alopecia	Oral glucocorticoids and azathioprine, subcutaneous adalimumab	OD: 20/60 OS: counting finger
Yepez et al.^25^	29/F	OD: 20/100 OS: 20/300	2 weeks	OU: optic nerve swelling, serous RD	Tinnitus	Methylprednisolone pulses (3 days), oral prednisolone 1 mg/kg/day	OD: 20/60 OS: 20/80
Anthony et al.^26^	23/F	OD: 20/30 OS: 20/40	3 weeks	OU: anterior uveitis, vitritis, disk hyperemia, serous RD, BLD	No	Oral prednisolone 1 mg/kg/day	OU: 20/25
Saraceno et al.^27^	37/F	OU: hand motion	2 Weeks	OD: anterior uveitis OU: vitritis, optic disk hyperemia, serous RD	Tinnitus	Oral prednisone 1 mg/kg/day	OD: 20/25 OS: 20/50

Abbreviations: BLD, bacillary layer detachment; F, female; Interval, duration between SARS‐CoV‐2 infection and onset of symptoms; M, male; RD, retinal detachment.

**Figure 4:**
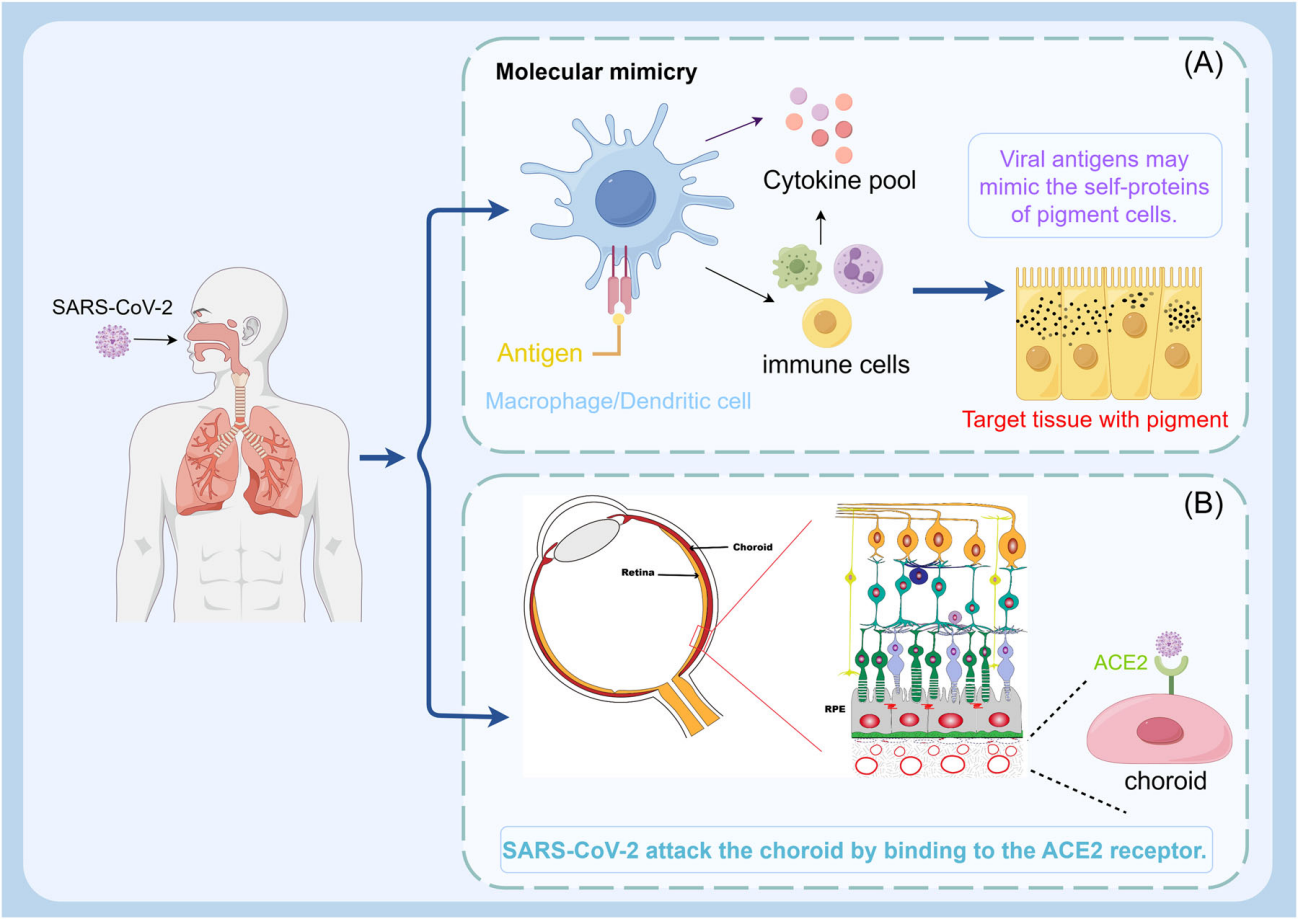
Schematic hypothesis for the mechanism of VKH disease following SARS‐CoV‐2 infection: (A). SARS‐CoV‐2 may provoke VKH disease through molecular mimicry. SARS‐CoV‐2 viral antigens may mimic the self‐proteins of pigment cells. Following the SARS‐CoV‐2 infection, cytokines and activated immune cells may form a complicated network of immune responses. Under the specific immune environment, activated effector T cells and other effector immune cells may attack pigmented tissue to initiate VKH disease. (B). SARS‐CoV‐2 infection may directly provoke VKH disease. The angiotensin‐converting enzyme 2 (ACE2) receptor is expressed in the choroid. SARS‐CoV‐2 may attack the choroid by binding to the ACE2 receptor. SARS‐CoV‐2, severe acute respiratory syndrome coronavirus 2.

With all these facts, the triggering role of COVID‐19 infection in the development of VKH disease is widely acknowledged but remains uncertain. Based on the analysis above, SARS‐CoV‐2 infection may directly provoke VKH disease and also potentially induce it through molecular mimicry. It is well‐established that SARS‐CoV‐2 enters host cells by binding to the angiotensin‐converting enzyme 2 (ACE2) receptor. Several researchers have confirmed that the ACE2 receptor is expressed in the eye, specifically on the conjunctiva, choroid, vascular endothelium, and nerves.^29^ Thus, it is proposed that SARS‐CoV‐2 may attack the choroid resulting in the development of VKH disease (Figure 4B). In addition, SARS‐CoV‐2 may cause dysfunction of immune responses characterized by lymphopenia and an activated lymphocyte profile or dysfunction.^30^ T cells not only possess some cell markers that may render them susceptible to VKH disease, but also produce a certain profile of cytokines that may have an effect on the differentiation of naïve T cells, thereby forming a complicated immune environment.^30^, ^31^ And, SARS‐CoV‐2 viral antigens may mimic the self‐proteins of pigment cells. So, under the specific immune environment, activated effector T cells and other effector immune cells may attack tissue with pigment, including the choroid, ear, skin, and meninges (Figure 4A). However, we need to provide additional insight into the definitive underlying mechanism by which SARS‐CoV‐2 infection initiates the onset of VKH disease.

This study has some limitations. Because of the high cost and limited availability of genetic testing, genetic testing was not performed in our patient. Consequently, we cannot confirm the genetic contribution to the pathogenesis of VKH disease. Furthermore, as a case report, the findings regarding the relationship between SARS‐CoV‐2 infection and VKH disease cannot be extrapolated to the broader population. Further studies are needed to reveal the exact pathogenesis that could be an aid to manage VKH disease. In light of these findings, it is crucial for ophthalmologists and physicians to be aware of the potential link between VKH disease and COVID‐19. Based on the studies reviewed above, it is plausible to consider the SARS‑CoV‑2 virus as a possible trigger for VKH disease.

## AUTHOR CONTRIBUTIONS

**Hui Zou**: Writing—original draft; writing—review and editing. **Ke Zhang**: Validation. **Xuan Chen**: Writing—review and editing. **Sha Sha**: Data curation; resources.

## Data Availability

The data that support the findings of this study are openly available.

## References

[1] Du L , Kijlstra A , Yang P . Vogt‐Koyanagi‐Harada disease: novel insights into pathophysiology, diagnosis and treatment. Prog Retinal Eye Res. 2016;52:84‐111. 10.1016/j.preteyeres.2016.02.002 26875727

[2] Fonollosa A , Hernández‐Rodríguez J , Cuadros C , et al. Characterizing COVID‐19‐related. Retina. 2022;42:465‐475. 10.1097/iae.0000000000003327 34914345

[3] Benito‐Pascual B , Gegúndez JA , Díaz‐Valle D , et al. Panuveitis and optic neuritis as a possible initial presentation of the novel coronavirus disease 2019 (COVID‐19. Ocul Immunol Inflamm. 2020;28:922‐925. 10.1080/09273948.2020.1792512 32870739

[4] Read RW , Holland GN , Rao NA , et al. Revised diagnostic criteria for Vogt‐Koyanagi‐Harada disease: report of an international committee on nomenclature. Am J Ophthalmol. 2001;131:647‐652. 10.1016/s0002-9394(01)00925-4 11336942

[5] Jiang H , Li Z , Yu L , et al. Corrigendum: immune phenotyping of patients with acute Vogt‐Koyanagi‐Harada syndrome before and after glucocorticoids therapy. Front Immunol. 2021;12:659150. 10.3389/fimmu.2021.659150 33995378 PMC8113950

[6] Joye A , Suhler E . Vogt‐Koyanagi‐Harada disease. Curr Opin Ophthalmol. 2021;32:574‐582. 10.1097/icu.0000000000000809 34545845

[7] Hotta Y , Hayakawa M , Kawano H , et al. Analysis of herpes virus group (DNA) from cerebrospinal fluid in vogt‐koyanagi‐harada disease. Ocul Immunol Inflamm. 1996;4:99‐103. 10.3109/09273949609079639 22827414

[8] Bassili SS , Peyman GA , Gebhardt BM , Daun M , Ganiban GJ , Rifai A . Detection of Epstein‐Barr virus DNA by polymerase chain reaction in the vitreous from a patient with Vogt‐Koyanagi‐Harada syndrome. Retina. 1996;16:160‐161. 10.1097/00006982-199616020-00013 8724962

[9] Sugita S , Takase H , Kawaguchi T , Taguchi C , Mochizuki M . Cross‐reaction between tyrosinase peptides and cytomegalovirus antigen by T cells from patients with Vogt‐Koyanagi‐Harada disease. Int Ophthalmol. 2007;27:87‐95. 10.1007/s10792-006-9020-y 17253112

[10] Sugita S , Takase H , Taguchi C , et al. Ocular infiltrating CD4+ T cells from patients with Vogt‐Koyanagi‐Harada disease recognize human melanocyte antigens. Invest Opthalmol Visual Sci. 2006;47:2547‐2554. 10.1167/iovs.05-1547 16723469

[11] Liang L , Tan X , Zhou Q , Tian Y , Kijlstra A , Yang P . TLR3 and TLR4 but not TLR2 are involved in Vogt‐Koyanagi‐ harada disease by triggering proinflammatory cytokines production through promoting the production of mitochondrial reactive oxygen species. Curr Mol Med. 2015;15:529‐542. 10.2174/1566524015666150731095611 26238371

[12] Wade CI , Earley KE , Justin GA , Weber ML . Vogt‐Koyanagi‐Harada disease presenting secondary to a post‐infectious *Mycoplasma pneumoniae* autoimmune response. Am J Ophthalmol Case Rep. 2020;19:100793. 10.1016/j.ajoc.2020.100793 32613142 PMC7320312

[13] Yoshino N , Kawamura A , Ishii A , et al. Vogt‐Koyanagi‐Harada disease associated with influenza A virus infection. Intern Med. 2018;57:1661‐1665. 10.2169/internalmedicine.9819-17 29321438 PMC6028674

[14] Iwasaki M , Saito J , Zhao H , Sakamoto A , Hirota K , Ma D . Inflammation triggered by SARS‐CoV‐2 and ACE2 augment drives multiple organ failure of severe COVID‐19: molecular mechanisms and implications. Inflammation. 2021;44:13‐34. 10.1007/s10753-020-01337-3 33029758 PMC7541099

[15] Huang C , Wang Y , Li X , et al. Clinical features of patients infected with 2019 novel coronavirus in wuhan, China. Lancet. 2020;395:497‐506. 10.1016/s0140-6736(20)30183-5 31986264 PMC7159299

[16] Erol MK , Bozdogan YC , Suren E , Gedik B . Treatment of a full‐thickness macular hole and retinal detachment secondary to toxoplasma chorioretinitis that developed shortly after COVID‐19: a case report. J Français d'Ophtalmol. 2022;45:446‐451. 10.1016/j.jfo.2021.12.004 PMC8733215 35034856

[17] Miyata M , Ooto S , Muraoka Y . Punctate inner choroidopathy immediately after COVID‐19 infection: a case report. BMC Ophthalmol. 2022;22:297. 10.1186/s12886-022-02514-8 35799141 PMC9260973

[18] Aggarwal K , Agarwal A , Jaiswal N , et al. Ocular surface manifestations of coronavirus disease 2019 (COVID‐19): a systematic review and meta‐analysis. PLoS One. 2020;15:e0241661. 10.1371/journal.pone.0241661 33151999 PMC7643964

[19] Somboonviboon D , Wattanathum A , Keorochana N , Wongchansom K . Sarcoidosis developing after COVID‐19: a case report. Respirol Case Rep. 2022;10:e01016. 10.1002/rcr2.1016 35978720 PMC9366406

[20] Ding X , Chang Q . Probable Vogt‐Koyanagi‐Harada disease after COVID‐19 vaccination: case report and literature review. Vaccines. 2022;10:783. 10.3390/vaccines10050783 35632539 PMC9146171

[21] de Queiroz Tavares Ferreira F , Araújo DC , de Albuquerque LM , Bianchini PM , Holanda EC , Pugliesi A . Possible association between Vogt‐Koyanagi‐Harada disease and coronavirus disease vaccine: A report of four cases. Ocul Immunol Inflamm. 2022;31:1134‐1140. 10.1080/09273948.2022.2093756 35914285

[22] Brunet de Courssou JB , Tisseyre M , Hadjadj J , et al. De novo Vogt‐Koyanagi‐Harada disease following Covid‐19 vaccine: a case report and literature overview. Ocul Immunol Inflamm. 2022;30:1292‐1295. 10.1080/09273948.2022.2028291 35113742

[23] Eatz T , Charles JH . Vogt‐Koyanagi‐Harada syndrome in the setting of COVID‐19 infection. Clin Case Rep. 2023;11:e6617. 10.1002/ccr3.6617 36950665 PMC10025255

[24] Santamaria A , Chang J , Savarain C . SARS‐CoV‐2 among the potential viral triggers for Vogt‐Konayagi‐Harada disease: first case report and literature review. Ocul Immunol Inflamm. 2022;30:1869‐1875. 10.1080/09273948.2021.1966052 34436960

[25] Yepez JB , Murati FA , Petitto M , et al. Vogt‐Koyanagi‐Harada disease following COVID‐19 infection. Case Rep Ophthalmol. 2021;12:804‐808. 10.1159/000518834 34720981 PMC8525290

[26] Anthony E , Rajamani A , Baskaran P , Rajendran A . Vogt koyanagi harada disease following a recent COVID‐19 infection. Indian J Ophthalmol. 2022;70:670‐672. 10.4103/ijo.IJO_2550_21 35086262 PMC9023905

[27] Saraceno JJF , Souza GM , dos Santos Finamor LP , Nascimento HM , Belfort R . Vogt‐Koyanagi‐Harada syndrome following COVID‐19 and ChAdOx1 nCoV‐19 (AZD1222) vaccine. Int J Retina Vitreous. 2021;7:49. 10.1186/s40942-021-00319-3 34462013 PMC8404022

[28] Dutta Majumder P , Sadhu S , González‐López JJ , Mochizuki M . A COVID‐19 perspective of Vogt‐Koyanagi‐Harada disease. Indian J Ophthalmol. 2023;71:2587‐2591. 10.4103/ijo.Ijo_172_23 37322685 PMC10417979

[29] Seah I , Agrawal R . Can the coronavirus disease 2019 (COVID‐19) affect the eyes? A review of coronaviruses and ocular implications in humans and animals. Ocul Immunol Inflamm. 2020;28:391‐395. 10.1080/09273948.2020.1738501 32175797 PMC7103678

[30] Silva LT , Ortega MM , Tiyo BT , et al. SARS‐CoV‐2 recombinant proteins stimulate distinct cellular and humoral immune response profiles in samples from COVID‐19 convalescent patients. Clinics. 2021;76:e3548. 10.6061/clinics/2021/e3548 34878034 PMC8610223

[31] Rabaan, AA , Al‐Ahmed SH , Garout MA , et al. Diverse immunological factors influencing pathogenesis in patients with COVID‐19: a review on viral dissemination, immunotherapeutic options to counter cytokine storm and inflammatory responses. Pathogens 10, 565, 10.3390/pathogens10050565 (2021). 34066983 PMC8150955

